# Pathogenicity and genetic characterisation of a novel reassortant, highly pathogenic avian influenza (HPAI) H5N6 virus isolated in Korea, 2017 

**DOI:** 10.2807/1560-7917.ES.2018.23.7.18-00045

**Published:** 2018-02-15

**Authors:** Young-Il Kim, Young-Jae Si, Hyeok-Il Kwon, Eun-Ha Kim, Su-Jin Park, Norbert John Robles, Hiep Dinh Nguyen, Min-Ah Yu, Kwang-Min Yu, Youn-Jeong Lee, Myoung-Heon Lee, Young Ki Choi

**Affiliations:** 1 College of Medicine and Medical Research Institute, Chungbuk National University, Seowon-gu, Cheongju, South Korea; 2 These authors contributed equally to this article; 3 Avian Influenza Research and Diagnostic Division, Animal and Plant Quarantine Agency, Gimcheon-si, Gyeongsangbuk-do, South Korea

**Keywords:** Influenza A virus, H5N6, Reassortant, Highly pathogenic avian influenza (HPAI), Virulence, South Korea

## Abstract

We investigated influenza A(H5N6) viruses from migratory birds in Chungnam and Gyeonggi Provinces, South Korea following a reported die-off of poultry in nearby provinces in November 2017. Genetic analysis and virulence studies in chickens and ducks identified our isolate from December 2017 as a novel highly pathogenic avian influenza virus. It resulted from reassortment between the highly virulent H5N8 strain from Korea with the N6 gene from a low-pathogenic H3N6 virus from the Netherlands.

In connection with an influenza A(H5N6) outbreak in poultry in November 2017 in JeollabukDo Province, South Korea [1], we collected influenza virus isolates investigated from wild migratory birds in the neighbouring Chungnam and Gyeonggi Provinces. Four novel reassortant highly pathogenic avian influenza (HPAI) H5N6 viruses were isolated on 13 December 2017 and continued to cause outbreaks in domestic poultry [personal communication: Dr Youn-Jeong Lee**,** Animal and Plant Quarantine Agency, South Korea, January 2018]. Genetic characterisation revealed that the haemagglutinin (HA) gene of the novel H5N6 viruses was closely associated with clade 2.3.4.4 influenza A(H5N8) viruses. Although genetically similar to H5N6 viruses that caused outbreaks in Japan (first reported on 10 November 2017) [2] and the Netherlands (7 December 2017) [3], there has not been a report about its pathogenic potential in poultry species. Therefore, we report here the genetic characterisation of the novel influenza A(H5N6) virus and the investigation of its pathogenic potential in chickens and ducks.

## Genetic characterisation of novel influenza A(H5N6) viruses

**Figure 1:**
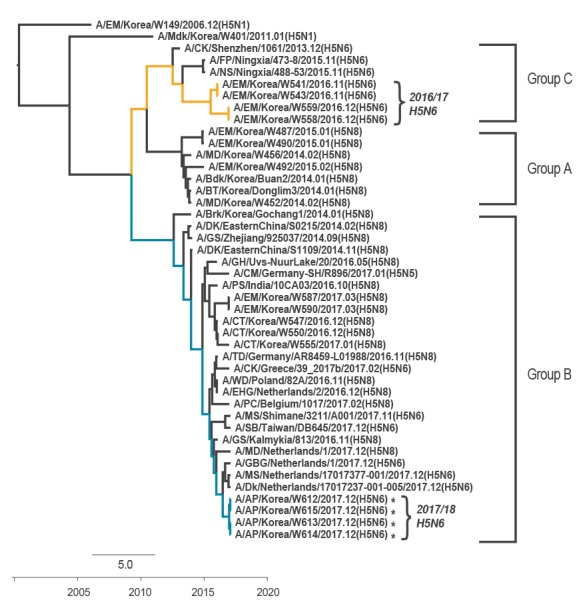
Phylogenetic trees comparing the haemagglutinin nucleotide sequences of 2017/18 Korean influenza A(H5N6) viruses, South Korea, December 2017 (n = 4)

**Figure 2:**
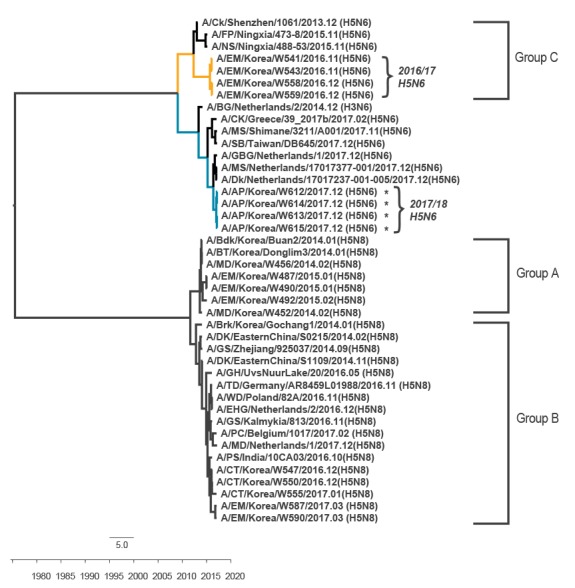
Phylogenetic trees comparing the neuraminidase nucleotide sequences of novel influenza A(H5N6) viruses, South Korea, December 2017 (n = 4)

Four influenza A(H5N6) viruses were isolated from faecal samples obtained from migratory bird habitats in Gyeonggi Province during a surveillance study conducted on 13 December 2017. Mitochondrial DNA sequence analysis of the faecal specimens revealed *Anas platyrhynchos* to be the viral host. In a full-length genomic sequence analysis, the viruses showed 99.9–100% nucleotide homology to one another but 97.2–99.4% homology with influenza A(H5N6) viruses from Japan and the Netherlands. Genetic and phylogenic analysis revealed that our strains clustered with the A/Tufted Duck/Germany/AR8459-L01988/2016(H5N8)-like viruses (clade 2.3.4.4 A/Brk/Korea/Gochang1/2014-like H5N8 lineage) (Figure 1), although their neuraminidase (NA) gene segments were derived from A/BG/Netherlands/2/2014(H3N6)-like viruses persisting in Europe during 2017 (Figure 2).

Bdk: broiler duck; Brk: breeder duck; BT: baikal teal; CK: chicken; CT: common teal; DK: duck; EHG: Eurasian herring gull; EM: environment; FP: ferruginous pochard; GBG: great black backed gull; GH: grey heron; GS: goose; HA: haemagglutinin; MD: mallard; Mdk: mallard duck; MS: mute swan; NS: northern shoveler; PC: peacock; PS: painted stork; RS: ruddy shelduck; SB: spoonbill; TD: tufted duck; WD: wild duck.

To investigate the origins of novel influenza A(H5N6) viruses (A/AP/Korea/W612/2017, A/AP/Korea/W613/2017, A/AP/Korea/W614/2017 and A/AP/Korea/W615/2017: marked with asterisks), full-length nucleotide sequences of HA genes were compared with available H5Nx and high blast-scoring virus sequences from GenBank and the Global Initiative on Sharing All Influenza Data (GISAID). The deposited GenBank accession numbers of HA genes are MG891804–MG891807; GISAID sequences are acknowledged in Supplement 1. Time-scaled phylogenies (dates shown on the horizontal axis) were inferred using strict-clock Bayesian Markov Chain Monte Carlo analysis. The month of isolation is indicated at the end of the virus name.

The yellow line indicates the 2016/17 influenza A(H5N6) subtypes while the blue line indicates the 2017/18 H5N6 subtype which was isolated from South Korea. The Group A of clade 2.3.4.4 viruses comprises influenza A(H5N8) viruses identified in South Korea in the 2013/14 and 2014/15 winter seasons. Group B comprises H5N8, H5N6 and H5N5 viruses identified in Asia and Europe during the 2013/14 to 2017/18 winter seasons. Group C of clade 2.3.4.4 viruses comprises H5N6 viruses identified in China and South Korea during the 2015/16 and 2017/18 winter seasons, respectively.

Bdk: broiler duck; BG: barnacle goose; Brk: breeder duck; BT: baikal teal; CK: chicken; CT: common teal; DK: duck; EHG: eurasian herring gull; EM: environment; FP: ferruginous pochard; GBG: great black backed gull; GH: grey heron; GS: goose; MD: mallard; Mdk: mallard duck; MS: mute swan; NA: neuraminidase; NS: northern shoveler; PC: peacock; PS: painted stork; RS: ruddy shelduck; SB: spoonbill; TD: tufted duck; WD: wild duck.

To investigate the origins of our novel influenza A(H5N6) viruses (A/AP/Korea/W612/2017, A/AP/Korea/W613/2017, A/AP/Korea/W614/2017 and A/AP/Korea/W615/2017: marked with asterisks), full-length nucleotide sequences of NA genes were compared with available H5Nx and high blast scoring virus sequences from the GenBank and Global Initiative on Sharing All Influenza Data (GISAID) databases; GISAID sequences are acknowledged in Supplement 1. The deposited GenBank accession numbers of NA genes are MG891812-MG891815.Time-scaled phylogenies (dates shown on the horizontal axis) were inferred using strict-clock Bayesian Markov Chain Monte Carlo analysis. The month of isolation is indicated at the end of the virus name.

The yellow line indicates the 2016/17 influenza A(H5N6) viruses while the blue line indicates the 2017/18 H5N6 viruses that were isolated from South Korea. The Group A of clade 2.3.4.4 viruses comprises influenza A(H5N8) viruses identified in South Korea in the 2013/14 and 2014/15 winter seasons. Group B comprises H5N8 and H5N6 viruses identified in Asia and Europe during the 2013/14 to 2017/18 winter seasons. Group C of clade 2.3.4.4 viruses comprises H5N6 viruses identified from China in the 2015/16 and South Korea in the 2016/17 winter seasons, respectively.

**Figure 3:**
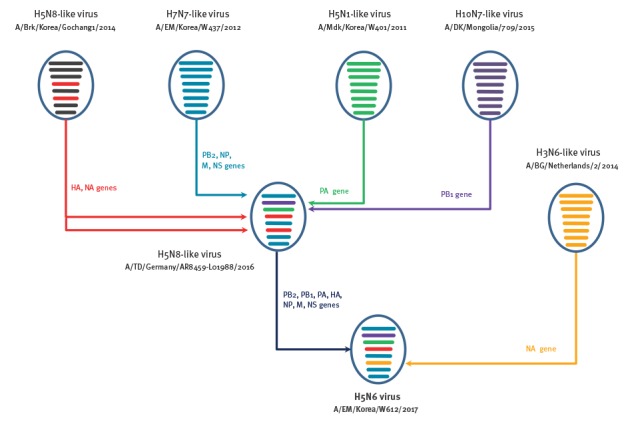
Genotype illustration of the reassortment process of 2017/18 Korean influenza A(H5N6) viruses, South Korea

BG: barnacle goose; Brk: breeder duck; DK: duck; EM: environment; HA: haemagglutinin; M: matrix; Mdk: mallad duck; NA: neuraminidase; NP: nucleoprotein; NS: nonstructural; PA: polymerase acidic; PB: polymerase basic; TD: tufted duck.

In contrast to the previous 2016/17 Korean influenza A(H5N6) viruses [4], the HA gene of 2017/18 Korean influenza A(H5N6) viruses did not belong to Group C of the clade 2.3.4.4 HPAI H5 viruses. Instead, they were closely associated with Group B of the clade 2.3.4.4 H5N8 viruses that have mainly been circulating in Eurasia [5]. Further, the PB2, NP, M and NS genes of 2017/18 Korean influenza A(H5N6) viruses were closely related to A/EM/Korea/W437/2012(H7N7)-like influenza strains, the PB1 gene was closely related to the A/DK/Mongolia/709/2015(H10N7)-like virus, while the PA genes were closely related to the A/EM/Korea/W401/2011(H5N1)-like virus and clustered together with the European influenza A(H5N8) viruses (A/TD/Germany/AR8459-L01988/2016-like viruses) (Figure 3).

The AP/W612/H5N6 virus is a reassortant of at least five different subtypes (H5N8, H7N7, H5N1, H10N7, and H3N6) from the natural gene pool in Eurasian avian influenza viruses. The eight gene segments in each illustrative virus particle represent (from top to bottom) the PB2, PB1, PA, HA, NP, NA, M and NS genes. Each colour represents a virus lineage. Black: origin from A/Brk/Korea/Gochang1/2014-like; blue: A/EM/Korea/W437/2012-like; purple: A/DK/Mongolia/709/2015-like; light green: A/Mdk/Korea/W401/2011-like; red: Clade 2.3.4.4 H5 HA and NA genes.

Molecular analysis demonstrated that the HA cleavage site of the 2017/18 Korean influenza A(H5N6) viruses contained polybasic residues (REKRRK/G), which denotes a high-pathogenicity phenotype in chickens. All four 2017/18 Korean influenza A(H5N6) viruses maintained the glutamine residue at position 226 (H3 numbering) and a glycine residue at position 228. While all influenza A(H5N6) strains isolated in Korea in 2016 had one amino acid deletion in their HA1 133 site, a mutation commonly found in the 2.3.4.4 HA genes of human infectious influenza A(H5N6) viruses [4], the 2017/18 Korean influenza A(H5N6) viruses do not contain this deletion (Table 1). In addition, the 2017/18 Korean influenza A(H5N6) viruses did not contain the characteristic amino acid deletion (position 49–68 bases) in the NA gene that is present in the 2016 influenza A(H5N6) virus. With the exception of the NS gene, the internal genes of 2017/18 Korean influenza A(H5N6) viruses displayed the same characteristics as the 2016/17 Korean influenza A(H5N8) viruses (Table 1).

**Table 1:** Molecular comparison of H5 avian influenza viruses and similar isolates, South Korea, December 2017 (n = 8)

Viruses^a^	HA clade	HA sequence (aa)									HA deletion	NA stalk deletion	NS1			PB2 sequence at aa		Expression of PB1-F2 protein
		Cleavage site	Receptor binding sites										Deletion of aa 80–84	aa residue at				
		335–348^b^	158	193	222	224	226	227	228	318	133	49–68		92	C-term	627	701	
**AP/Korea/W612/17**	**2.3.4.4**	**REKRRK__ /G**	**N**	**N**	**Q**	**N**	**Q**	**R**	**G**	**T**	**No**	**No**	**No**	**D**	**GSEV**	**E**	**D**	**Yes**
PC/Belgium/1017/17	2.3.4.4	REKRRK__ /G	N	N	Q	N	Q	R	G	T	No	No	No	D	GSEV	E	D	Yes
TD/Germany/AR8459-L01988/16	2.3.4.4	REKRRK__ /G	N	N	Q	N	Q	R	G	T	No	No	No	D	GSEV	E	D	Yes
EM/Korea/W541/16	2.3.4.4	RERRRK__/G	N	N	Q	N	Q	Q	G	T	Yes	Yes	Yes	E	ESEV	E	D	Yes
CT/Korea/W555/17	2.3.4.4	REKRRK__ /G	N	N	Q	N	Q	R	G	T	No	No	No	D	GSEV	E	D	Yes
MD/Korea/W452/14	2.3.4.4	RERRRK__/G	N	N	Q	N	Q	R	G	T	No	No	No	D	ESEVRG	E	D	Yes
BDk/Korea/Gochang1/14	2.3.4.6	REKRRK __/G	N	N	Q	N	Q	R	G	T	No	No	No	D	ESEV	E	D	Yes
EM/Korea/W149/06	2.2	GERRRKKR/G	N	K	K	N	Q	S	G	T	No	Yes	Yes	D	ESKV	K	D	Yes

aa: amino acid; AP: *Anas platyrhynchos*; BDk: breeder duck; CT: common teal; C-term: 4 aa sequence at the C-terminal end; EM: environment; HA: haemaggutinin; MD: mallard duck; NA: neuraminidase; PC: peacock; RBS: receptor binding site; TD: tufted duck.

^a^ The isolate in bold was the 2017 Korean highly pathogenic avian influenza H5N6 viruses examined in this study.

^b^ H3 numbering.

## Virulence in chickens and ducks

One representative virus, A/AP/Korea/W612/2017(H5N6), was selected for further study. The intravenous pathogenicity index (IVPI) was measured in accordance with World Organisation for Animal Health (OIE) standards. In chickens, the IVPI score was 2.76, resulting in classification of the A/AP/Korea/W612/2017(H5N6) virus as HPAI [6]. To measure the chicken and duck lethal dose 50% (CLD_50_ and DLD_50_), specific pathogen-free chickens and ducks were infected with 10^7^–10^2^ egg infectious dose (EID)_50_/mL by intranasal inoculation. The CLD_50_ was 2.83 log_10_EID_50_/mL, with which the chickens died within 3 to 5 days after infection. In contrast, none of the infected ducks died during the experimental period (14 days) suggesting a DLD_50_ of more than 10^7^ EID_50_/mL.

To investigate the pathogenicity and horizontal transmission ability of the A/AP/Korea/W612/2017(H5N6) virus in chickens and ducks, we proceeded with a group of intranasally inoculated (10^6^ EID_50_/mL) animals (n = 14) and added a direct contact group (n = 3) one day after infection. Oropharyngeal and cloacal swab samples were collected for virus titration for 14 days post infection (dpi). Chickens in the infection and direct contact groups all succumbed by 4 dpi and 5 dpi, respectively. Moreover, virus was detected at all time points of swab collection and peaked at 2 dpi at 4.8 log_10_ EID_50_/mL in oropharyngeal swabs (Table 2). In the direct contact group, the virus was detected from the second day after contact and the highest cloacal swab viral titre (5.6 log_10_ EID_50_/mL) was measured on the fourth day. To determine virus tissue distribution of the A/AP/Korea/W612/2017(H5N6) virus in infected chickens, we collected the lung, brain, kidney, spleen, heart, liver and colon from three birds each at 3 and 5 dpi using individual sterile equipment to avoid cross-contamination between them. The virus was detected in all organs collected from the inoculated chicken group at 3 dpi, the last point at which samples could be collected due to the lethality of the virus (Table 2).

**Table 2:** Viral titres in chickens and ducks experimentally inoculated with influenza A/AP/Korea/W612/2017(H5N6), South Korea, December 2017 (n = 17)

Experimental setup		Chicken					Duck								Seroconversion (HI GMT)
Infected group: swab^a^	Time point	1 dpi	2 dpi	3 dpi	4 dpi^c^		1 dpi		3 dpi		5 dpi		7 dpi	9 dpi	640
	Oropharyngeal	2.7 ± 0.4	4.8 ± 0.5	4.1 ± 0.4	NC		3.0 ± 0.3		5.1 ± 0.2		4.9 ± 0.4		1.9 ± 0.4	ND	
	Cloacal	1.1 ± 0.4	3.1 ± 0.4	3.2 ± 0.2	NC		1.0 ± 0.4		1.8 ± 0.5		1.3 ± 0.3		ND	ND	
Contact group: swab^a^	Time point	1 dpc	2 dpc	3 dpc	4 dpc	5 dpc^c^	1 dpc	2 dpc	3 dpc	4 dpc	5 dpc	6 dpc	7 dpc		320
	Oropharyngeal	ND	1.3 ± 0.1	3.1 ± 0.5	4.2 ± 0.2	NC	1.1 ± 0.2	1.2 ± 0.3	4.7 ± 0.4	4.5 ± 0.4	3.8 ± 0.5	2.8 ± 0.5	ND		
	Cloacal	ND	1.1 ± 0.2	4.0 ± 0.5	5.6 ± 0.4	NC	ND	0.8 ± 0.5	1.9 ± 0.5	2.4 ± 0.4	2.2 ± 0.3	1.0 ± 0.2	ND		
Infected group: tissue^b^	Time point			3 dpi	4 dpi^c^				3 dpi		5 dpi				
	Lung			4.3 ± 0.1	NC				4.8 ± 0.5		4.0 ± 0.2				
	Brain			1.3 ± 0.1	NC				ND		ND				
	Kidney			3.8 ± 0.4	NC				5.1 ± 0.3		4.3 ± 0.1				
	Spleen			4.4 ± 0.4	NC				3.9 ± 0.5		2.5 ± 0.4				
	Heart			3.5 ± 0.4	NC				5.0 ± 0.5		3.0 ± 0.5				
	Liver			4.2 ± 0.3	NC				4.2 ± 0.3		1.3 ± 0.1				
	Colon			3.2 ± 0.3	NC				5.1 ± 0.2		2.0 ± 0.5				

dpc: days post contact; dpi: days post infection; GMT: geometric mean titre; HI: haemagglutination inhibition; NC: samples not collected; ND: none detected.

^a^ Viral titres are expressed as mean log_10_EID_50_/mL.

^b^ Viral titres are expressed as mean log_10_EID_50_/g.

^c^ Samples were not collected because chickens were all dead.

Titres are indicated with standard deviation. Swabs from chickens were collected daily for the infected and contact group. Swabs from ducks were collected every 2 days for the infected group and daily for the contact group.

In ducks, we measured the viral replication and transmission efficacy although no mortalities were observed. However, high viral titres were detected at all time points in oropharyngeal swabs of both groups, peaking at 3 dpi (5.1 log_10_ EID_50_/mL) for the infection group (Table 2). In contrast, low titres were observed in the cloacal swabs, which peaked at only 2.4 log_10_ EID_50_/mL at 4 dpi in the direct contact group. Furthermore, the virus was not detectable in cloacal swabs at 7 dpi or in cloacal or oropharyngeal swabs at 9 dpi for the inoculated group. For the contact group, the virus was not detected in oropharyngeal or cloacal swabs at 7 days post contact. Seroconversion was observed in all ducks inoculated with the A/AP/Korea/W612/2017(H5N6) virus and in their contact ducks, and haemagglutination inhibition geometric mean titres were 640 and 320 in the inoculated and direct contact group, respectively (Table 2). To investigate the tissue distribution of A/AP/Korea/W612/2017(H5N6) virus in ducks, we collected the lung, brain, kidney, spleen, heart, liver and colon from three birds each at 3 and 5 dpi. The A/AP/Korea/W612/2017(H5N6) virus was detected in all organs harvested from ducks in this experiment, except for the brain (Table 2).

## Discussion

In contrast to influenza A(H5N8) viruses, the clade 2.3.4.4 H5N6 virus first emerged in China in 2013 and became established in Asian countries such as Laos, Vietnam and mainland China [7-9] before causing large outbreaks in 2016 [4,10]. During the 2016/17 winter season, South Korea experienced large outbreaks of two different HPAI strains (H5N6 and H5N8) and as a result destroyed 640 million poultry (almost 30% of poultry in South Korea) [10-12]. 

In this study, we report the identification of a novel reassortant HPAI H5N6 virus that caused major outbreaks in domestic poultry during the 2017/18 winter season. This H5N6 virus is a reassortment of multiple subtypes (H5N8, H7N7, H5N1, H10N7 and H3N6) in the Eurasian gene pool of avian influenza viruses. Animal studies revealed that this novel influenza A(H5N6) virus is highly pathogenic in chickens but has attenuated virulence in ducks, which may result in their function as a virus reservoir. With the high susceptibility but attenuated virulence of HPAI infections in ducks, the duck can also be used as a sentinel for avian influenza virus surveillances [13]. Since the first avian influenza A(H5N8) (clade 2.3.4.4) virus was reported in Korean poultry in 2014 [14,15] and has continued to spread in South Korea, migratory birds have spread the virus to wild birds worldwide, including in Europe [16,17] and North America [18]. This rapid and widespread proliferation and reassortment underscores the need for continuous monitoring of avian influenza viruses in wild migratory birds, including virulence and pathogenicity studies using laboratory animals.
